# Evaluation of Retention and Fracture Strength of All Ceramic Crowns with Three Different Esthetic Cast Post–Core Systems

**DOI:** 10.1155/2023/6664894

**Published:** 2023-09-30

**Authors:** Sina Safari, Amirmohammad Mirzapour, Roya Sadrmohammadi

**Affiliations:** ^1^Student Research Committee and Department of Prosthodontics, School of Dentistry, Kerman University of Medical Sciences, Kerman, Iran; ^2^Dental Surgery, Kerman, Iran; ^3^Department of Prosthodontics, School of Dentistry, Kerman University of Medical Sciences, Kerman, Iran

## Abstract

**Purpose:**

This study investigates the fracture and retention strength of all-ceramic crowns with modified composite resin and ceramic cores compared to conventional casted post and core systems.

**Materials and Methods:**

A prepared human central tooth was initially scanned to design and 3D print the post and core. Subsequently, 40 bovine teeth were adjusted to accommodate the fabricated post and cores. They were then divided into four groups of 10 each: group 1 comprised cast cores without cover (control group), group 2 involved cast cores reduced and replaced with IPS Empress material (IPS group), group 3 consisted of cast cores covered with opaque composite (composite group), and group 4 included cast cores covered with opaque ceramic (ceramic group). Zirconia crowns were cemented onto all samples. After an aging process, pull-off and fracture strength tests were conducted. Fracture strength was determined by applying a compressive force at an angle of 135° to the tooth's longitudinal axis until the fracture occurred. For retention strength assessment, a universal testing machine with a 10 mm/min crosshead speed was employed. The resulting data underwent statistical analysis utilizing two-way analysis of variance (ANOVA) and Mann–Whitney *U* tests.

**Results:**

The analysis revealed no significant difference in fracture strength among the groups (*P*-value = 0.997). However, the retention strength of the control and IPS groups was significantly higher than that of the other groups.

**Conclusion:**

There were no discernible distinctions among the three study methods regarding fracture strength. Nonetheless, the retention strength of the IPS group resembled that of the control group, surpassing that of the composite and ceramic groups.

## 1. Introduction

The restoration of teeth following root canal treatment poses a substantial challenge in dentistry, owing to the considerable loss of crown structure that often results in structural weakness [1]. Metal–ceramic restorations have proven to be successful in dentistry due to their high fracture resistance. However, achieving natural translucency in these restorations is more intricate than all-ceramic counterparts, primarily due to the hindrance posed by the metal core, which obstructs light transmission in metal–ceramic restorations [2]. This characteristic of metal–ceramic restorations has consequently increased the utilization of all-ceramic alternatives [3].

Among the realm of translucent glass–ceramic materials, lithium disilicate has garnered popularity in the fabrication of both anterior and posterior restorations. This popularity stems from its superior esthetic qualities, commendable strength, erosion resistance, and chemical durability [4]. Nevertheless, the translucent cores of all-ceramic restorations, including lithium disilicate ceramics and translucent zirconia, are not suitable for concealing severely discolored teeth, titanium abutments, and metal-based posts and cores due to their adverse impact on the optical attributes of the final restoration [5]. In cases involving extensively damaged teeth, it is imperative to utilize posts to ensure the retention of restorations. Consequently, a diverse range of zirconia, fiber, and cast posts has been developed for this purpose. While zirconia and fiber posts are recommended due to their lesser influence on the optical properties of all-ceramic restorations, it is noteworthy that each post harbors distinct disadvantages [6].

To mitigate the impact of post and core color on the optical characteristics of all-ceramic restorations, studies have proposed the adoption of esthetic posts and cores, exemplified by zirconia posts with ceramic, fiber, or composite cores [7]. Furthermore, the recommendation includes using opaque materials to encapsulate metal posts and cores, such as opaque ceramic, opaque composite resin, and heat-pressed opaque porcelain (applied to the metal core). Alongside this, the utilization of restorations featuring opaque ceramic copings (e.g., polycrystalline ceramics) is advised. Such materials exhibit reduced transparency, possessing reflective and absorptive properties that curtail light penetration, thereby mitigating susceptibility to background influences [8].

This study aims to address the existing knowledge gap regarding concealed cast posts and cores' efficacy in enhancing the strength of the post, core, and all-ceramic crown assembly while promoting light transmission. Despite numerous recommendations supporting their incorporation, comprehensive data on their effectiveness are lacking. Additionally, crucial physical attributes like retention and fracture strength, potentially influenced by modifications to cast cores, have been overlooked in previous research. To fill this gap, the present study investigates and compares three distinct post and core systems: cores enveloped with IPS Empress, opaque composite resin, and ceramic with uncovered cast post and core. The primary objective is to identify the optimal system based on physical attributes beyond esthetics. The study also challenges the null hypothesis, exploring whether varying core modification methods impact fracture strength and retention properties of all-ceramic crowns on modified cores.

## 2. Materials and Methods

Initially, a sound human maxillary central tooth devoid of caries was mounted and underwent tooth preparation for zirconia full crowns. The incisal and axial surfaces were reduced by 1.5 mm. This trimming was conducted utilizing a 6° taper tool and a deep Chamfer finish line featuring a round internal angle. This procedure was facilitated using a high-speed handpiece equipped with a medium-grit diamond bur [9]. The prepared tooth crown was scanned for core design, and a suitable 10-mm long post was designed using the EXOCAD program [10]. Subsequently, 40 resin post and core samples were fabricated via a 3D printer (Flashforge Hunter, Jinhua, China), and they were then divided into four groups of 10 each.

### 2.1. Preparation of Dentin and Post and Core

Bovine teeth were employed as the study tooth samples, aligned with produced posts and cores. These bovine teeth were extracted from a bovine jaw before freezing, followed by immersion in a diluted sodium hypochlorite solution (5%) for 10 min. After thorough washing, the teeth were immersed in a water–alcohol solution. These teeth were subsequently sectioned into 14-mm long segments. After that, the dental canals were widened using piezo drills no. 2 and 3 (Mani, Tochigi, Japan) to match the post dimensions. The dentin was contoured to the core diameter, and a reduction was executed to achieve a 2-mm height of dentin for the ferrule. The remaining portion of the dentin was enveloped with a cold-cure acrylic material (Acropars 200; Marlic Co., Iran) along its vertical axis, using a surveyor [11].

#### 2.1.1. Preparation of the Control Group

The resin posts were cast with nickel–chromium alloy.

#### 2.1.2. Preparation of the IPS Group

A transparent silicone mold (Kristal A70, Muller-Omicron GmbH, Germany) was initially fashioned from the prepared tooth. The core section of the cast post and core underwent further reduction to align with the post diameter, achieved through bur manipulation. A thin layer of A3-shade opaque ceramic was administered to the core segment's surface, and the Duralay core was produced utilizing a silicone mold and cold-cure Duralay inlay pattern resin molds (Pattern Resin LS; GC America, Inc., USA). This process proceeded with the preparation of the all-ceramic restoration, subsequently the creation of the cast post with resin core for porcelain injection (IPS InLine PoM, Ivoclar Vivadent Inc., Liechtenstein). A heat pressing was performed using a furnace (VITA VACUMAT 6000 MP, Germany) [12].

#### 2.1.3. Preparation of the Composite Group

Following manufacturer guidelines, the resin core was initially reduced by half the diameter of a 008 bur on its buccal and proximal sides. This reduction was executed to facilitate the requisite space for composite opaque thickness, essential for masking metal color (0.4 mm). Following the reduction, casting was carried out, and adaptation to the dentin was assessed. Subsequently, all-ceramic restoration preparation ensued. Metal primer (MKZ Primer, Bredent GmbH, Senden, Germany) was then applied to the buccal and proximal sides of the core, corresponding to the reduced opaque composite, followed by the application of ∼0.2 mm of A3-shade composite opaque (Crealign Opaker, Bredent GmbH, Senden, Germany). This composite layer was subjected to a light curing device (ILED, Woodpecker, China) for 180 s, and a second 0.2-mm thick layer was similarly applied and cured [13].

#### 2.1.4. Preparation of the Ceramic Group

**Figure 1:**
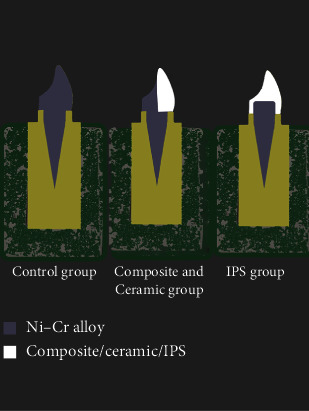
Schematic view of prepared posts and cores.

Initial reduction of the resin core by half the diameter of a 008 bur on the buccal and proximal surfaces was performed to create space for the thickness of opaque ceramic (ranging from 0.3 to 0.5 mm), which was required to conceal metallic color. Furthermore, subsequent casting and evaluation of adaptation to the dentin were carried out. Consequent stages encompassed all-ceramic restoration preparation, culminating in applying A3 ceramic opaque (Super Porcelain EX-3, Kuraray Noritake Dental Inc., Japan) to cover the core. Sintering was conducted using a furnace (VITA VACUMAT 6000 MP, Germany) [14] (Figure 1).

To ensure consistent cement thickness, light-body silicone was used to check post adaptation. The thickness of the silicone layer was examined under a microscope at two points: the cervical and apical areas and any dentin washes with a thickness other than 0.5 mm were removed and replaced. After confirming coordination between all posts and root canal dentin, self-cured glass ionomer cement (Luting & Lining Cement, GC, Tokyo, Japan) was utilized for cementation. Following a 24-hr interval, preparations for zirconia crowns were made [15].

### 2.2. Zirconia Full Crown Manufacturing and Porcelain Layering

Upon sample preparation, scanning was undertaken through a 3D dental scanner (Imetric 4D Imaging GmbH, Switzerland). Subsequent design and milling of zirconia coping, possessing a 65-*µ*m gap and 0.5-mm thickness, were executed [16] employing the Ceramill Mikro device (Amann Girrbach GmbH, Pforzheim, Germany). The control group involved high-opacity zirconia, while zirconia with high translucency (Bloomden Bioceramics Co., China) was employed for the study groups [17]. Post milling, a brief immersion in A3 dye (X-Color, XTCERA, China) for a duration of 5 s, was performed. Sintering was then conducted at a maximum temperature of 1,520°C for a span of 12 hr within a sintering furnace (Zirkonofen 600, Zirkonzahn, Italy). Subsequently, a layer of A3-shade porcelain (BOAT Co., China) was layered and sintered using a furnace (VITA VACUMAT 6000 MP, Germany) [18].

### 2.3. Crown Cementation

Post zirconia crown preparation, tooth surfaces underwent initial etching using 32% phosphoric acid for 15 s, followed by application of primers (Bis-Silane; BISCO Inc., USA). Subsequently, translucent cement (light-cured Choice2 Veneer Cement, BISCO Inc., USA) was employed for cementation. A final step encompassed curing each sample utilizing a light curing system for a duration of 40 s [19].

### 2.4. Fracture and Pull-Off Test

Prior to placing the samples into the testing apparatus, each group was subdivided into two subgroups of five for the fracture and retention strength assessments. The samples were maintained at 37°C for 24 hr, thereafter exposed to 5,000 thermal cycles ranging between 5 and 55°C (TC-300, Iran), each cycle involving a 30-s dwell time, to simulate artificial aging and replicate the oral environment [20].

During this study, the fracture and retention strengths of the posts, cores, and crowns were measured in Newtons utilizing the Instron test device.

#### 2.4.1. Pull-Off Test Preparation

**Figure 2:**
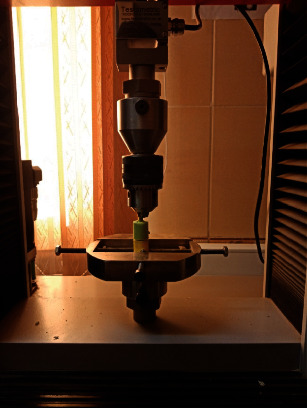
Pull-off test of samples with a universal testing machine.

For the pull-off test, the crown design incorporated protrusions to enhance retention within the acrylic part connected to the device. Acrylic material was applied along the longitudinal axis of the crowns, and alignment was secured using a screw affixed to the device head [20] (Figure 2).

#### 2.4.2. Fracture Strength Test

**Figure 3:**
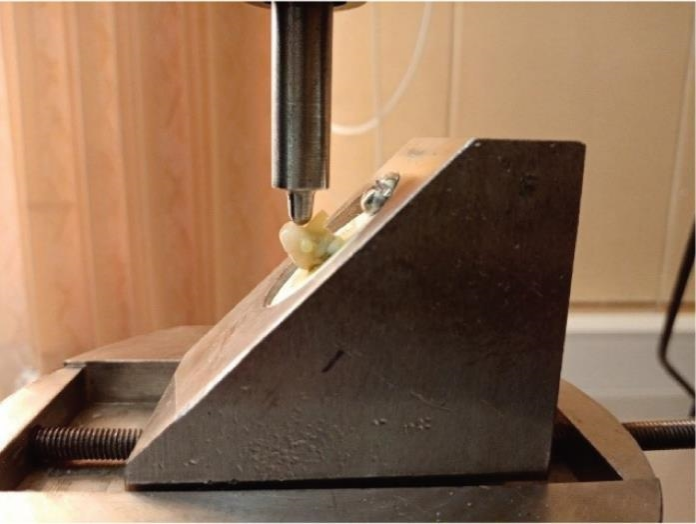
Fracture strength test of samples with universal testing machine.

A 4-mm diameter stainless steel bar was affixed to the device tip for the fracture strength test. A force was applied to the palatal surface at an angle of 135° relative to the tooth's longitudinal axis, with a speed of 0.5 mm/min and a distance of 4 mm from the incisal edge of the crown. This application continued until audible fracture evidence or visible changes on the monitor were detected [21, 22] (Figure 3).

### 2.5. Data Analysis

Data are expressed as mean ± standard deviation (SD) for numeric variables and are summarized by absolute frequencies and percentages for categorical variables. One-way analysis of variance (ANOVA) was used to compare the mean values of fracture and retentive strength across the studied groups. Tukey's multiple comparisons test was performed when a significance result observed in the one-way ANOVA. Normality assumption was not violated according to the nonparametric Kolmogorov–Smirnov test (*P* > 0.05). Homogeneity of variances presumption was also met according to the Levene's test (*P* > 0.05). Categorical variables were compared using Fisher's exact test among the groups. Statistical analysis was performed using SPSS software version 22 (IBM Corp., Armonk, NY). All *P*-values were two-tailed and considered significant at *P* ≤ 0.05.

## 3. Results

Table 1 provides the groups' mean and standard deviation of fracture strength. ANOVA analysis revealed no significant differences among the groups (*P*-value = 0.997) (Table 1).

The mean and standard deviation of retention strength across all groups are presented in Table 1. Pairwise comparisons indicated no significant differences between the control and IPS groups (*P*-value = 0.989) and similarly between the composite and ceramic group (*P*-value = 0.067). Nevertheless, significant differences were observed between the control and ceramic group (*P*-value = 0.006), control and composite group (*P*-value = 0.0001), IPS and ceramic group (*P*-value = 0.012), and IPS and composite group (*P*-value = 0.0001).

**Figure 4:**
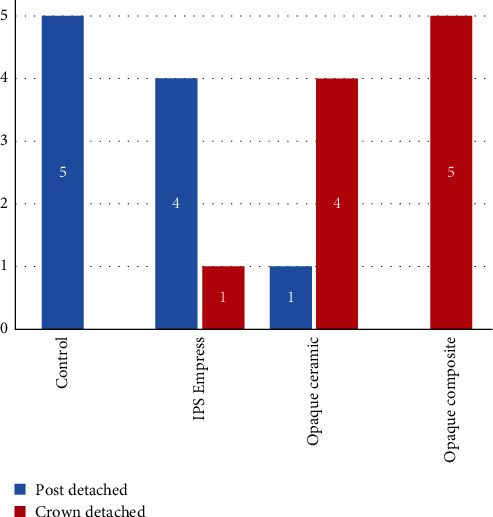
Comparison of the mode of failure among the group's Fisher's exact test; *P*-value = 0.003.

Analysis of failure mode during the pull-off test revealed that all posts detached from dentin in the control group. For the IPS group, detachment from dentin occurred in four samples, while one sample exhibited separation between the crown and core. In the ceramic group, post detachment occurred in one sample, whereas four samples experienced crown detachment from the core. In the composite group, crown detachment from the core was observed in all samples. Fisher's exact test indicated the significance of this phenomenon (*P*-value = 0.003) (Figure 4).

**Figure 5:**
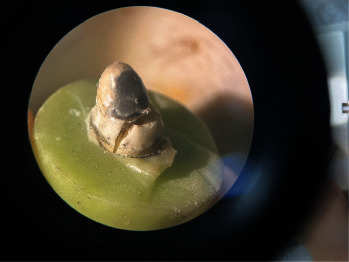
Fracture occured within 2 mm of cervical root dentin and considered as restorable.

**Figure 6:**
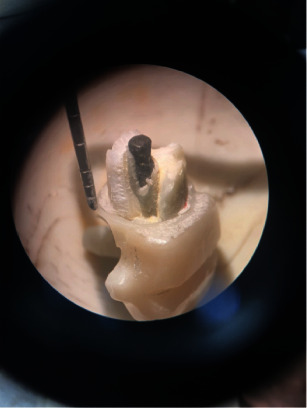
Fracture occured beyond 2 mm of cervical root dentin and considerable as nonrestorable.

**Figure 7:**
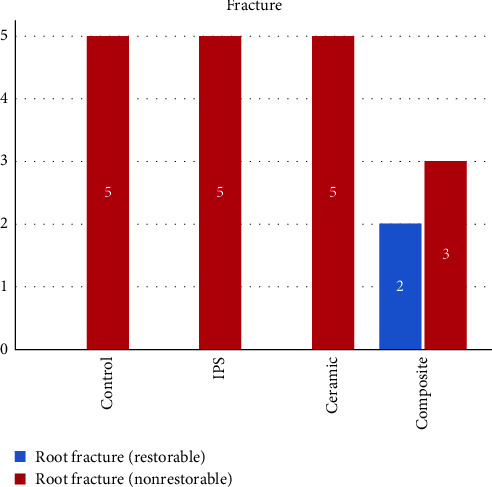
Comparison of root fracture among the studied groups. Fisher's exact test; *P*=0.211.

No fractures were found at the crown, core, or post–core interfaces in any of the samples. All fractures occurred within the dentin across all groups. Most of the fractures occurred within 2 mm of the cervical root dentin, allowing the tooth to be restored (Figure 5). However, in two samples of the composite group, the dentin fracture occurred beyond two cervical millimeters of cervical root dentin, rendering the tooth nonrestorable (Figure 6). A pairwise comparison of the mode of failure during the fracture test was conducted using the Mann–Whitney *U* test, and the relative frequency of restorable or nonrestorable teeth in the studied groups did not show a statistically significant difference (Figure 7).

## 4. Discussion

This study investigated the fracture and retention strength of cast posts and cores covered with composite resin, ceramic, and all-ceramic crowns in bovine teeth. The findings indicated that modifying the nickel–chromium core with ceramic and composite resin led to significant alterations in the retention of the post and core to the crowns. However, various groups had no significant differences in the fracture strength of the all-ceramic crowns and tooth assembly.

This study focused on examining the fracture strength of crowns and modified cores rather than the strength of the posts and teeth. Standardizing the samples minimizes the influence of variable parameters in the root canal (a 10-mm post within bovine dentin), enabling a specific examination of crown and core behaviors under compression force. The behavior of all groups was similar until dentin fracture occurred, and no fractures were observed in the modified crowns and cores, which served as the variable parameters.

Factors affecting tooth fracture strength include tooth crowning, remaining tooth structure, ferrule height, force magnitude and direction, post preparation materials, and bonding systems [23]. Since these variables were controlled in this study, they did not significantly affect the fracture strength among the investigated groups.

Fracture strength did not show significant differences between the IPS and control groups. Comparative studies on zirconia-composite and zirconia-ceramic post and core systems indicated that ceramic cores exhibited higher fracture resistance than composite cores [24]. For instance, Butz et al. [25] demonstrated that a zirconia post with a composite core had lower fracture strength compared to a zirconia post and core, cast post and core, and titanium post and composite core. The fracture strength of the cast post and core (426 N) was consistent with our findings [23]. Instead of constructing a composite core, the color of the metal core can be masked with an opaque composite layer without compromising its strength.

In the ceramic and composite groups, covering only the buccal and proximal surfaces of the core with thin layers of these materials did not significantly affect fracture strength, as the fracture strength of these two groups was similar to the control group. The study by Ozcan and Sahin [26] also found no significant differences in fracture strength among zirconia posts with zirconia, feldspathic, and IPS Empress cores. Thus, alternative core options can be used instead of the costly IPS Empress core, based on characteristics other than fracture strength. The present study demonstrated that modifying the nickel–chromium core with ceramic and composite resin significantly altered the retention strength of the composite and ceramic groups compared to the control group. However, no significant difference was observed between the IPS and control groups.

Furthermore, comparisons in this study revealed that covering parts or the entire core with tooth-colored materials impacted crown retention. Although the retention values of the nickel–chromium cast core and the entire IPS Empress-covered core were not significantly different, it was evident that covering the post and core with a layer of opaque ceramic or composite could substantially reduce retention strength. While the ceramic group exhibited slightly lower mean retention than the composite group, the difference was not considered significant.

Various in vitro studies have explored post retention in different systems, considering variables such as post length, diameter, design, canal shape, preparation, luting material, cementing method, and location in the dental arch [27]. In the current study, these variables were consistent among groups, indicating that any differences in retention strength were attributed to the core variations.

The findings align with previous research, highlighting the significance of the dowel-core surface as a key variable in retention [27]. Roughening dentin walls or creating undercuts in the root canal walls have been shown to increase the retention of fiber posts significantly [28, 29]. Khaledi et al. [30] demonstrated that nickel–chromium and nonprecious gold alloy cast post and core systems exhibited similar retentive strength, with the cast nickel–chromium post and core closely matching the present study's results. Similarly, no fractures occurred in the core or at the post–core interface.

Retention comparisons between the control and IPS groups in the present study showed no significant difference. Moreover, no separation occurred between the post and core or from the covering material in the other study groups, which emphasizes the role of the core surface in crown retention. Crown retention was lower when only part of the core was covered with ceramic or composite compared to complete coverage with a ceramic coating (IPS Empress material), evident in both retention strength and mode of failure.

Several studies have explored the impact of cement on the retention of all-ceramic crowns, suggesting that resin cement provides stronger bonding in crowns with IPS Empress ceramic cores compared to composite cores [31, 32]. In the present study, the same resin cement was used across all groups, and teeth preparation for cementing followed Choice 2 cement instructions for dentin. No additional treatment was applied to the opaque composite or ceramic surfaces, focusing on studying group behavior without external interventions.

During thermocycling and temperature changes, the stress on the cement likely differed among groups. The cement in the composite and ceramic groups experienced more stress due to its contact with three materials with distinct thermal expansion coefficients, whereas in the control and IPS groups, the cement interacted with only two materials. Future studies could consider covering the entire core surface with opaque composite or ceramic to minimize stress resulting from differences in thermal expansion coefficients.

In conclusion, achieving both retention and fracture strength is essential when employing post and core systems. However, enhancing retention often involves the removal of tooth structure, potentially compromising root strength. Dentists should individually evaluate each tooth to determine the optimal approach for balancing maximum fracture strength and retention. Since a single-post system may not universally meet retention requirements, different post systems aim to balance post retention and root fracture strength. Such a flexible approach enables the successful reconstruction of root-treated teeth by selecting a post system that provides necessary retention while minimizing the risk of root strength reduction.

Future studies could employ a chewing simulator for sample preparation to simulate the clinical situation better. Moreover, larger sample sizes in laboratory studies and prospective clinical studies with follow-up periods could provide valuable insights into the effectiveness of these construction methods for root posts.

## 5. Conclusion

In conclusion, the study found no notable differences in fracture strength across the groups, as fractures consistently emerged within the dentin. While the retention strength showed some variation among specific group pairs, the analysis of failure modes during the pull-off test showed greater debonding tendencies in the partial-covered cores with composite and ceramic. These findings contribute to our grasp of dental materials' behavior, carrying important implications for real-world usage.

## Figures and Tables

**Table 1:** Comparison of fracture and retention strength means across the groups.

Group variable	Control (*n* = 5) mean ± SD (range)	IPS (*n* = 5) mean ± SD (range)	Ceramic (*n* = 5) mean ± SD (range)	Composite (*n* = 5) mean ± SD (range)	*P*-value^*∗*^
Fracture strength (*N*)	437.54 ± 70.14 (350.5–516.9)	442.76 ± 72.71 (380.7–559.0)	436.08 ± 45.19 (394.0–512.0)	433.98 ± 75.68 (362.0–552.0)	0.997

Retention strength (*N*)	178.20 ± 21.03^a^ (156–208)	173.60 ± 23.26^a^ (145–207)	121.40 ± 13.33^b^ (110–144)	82.20 ± 30.66^b^ (45–208)	<0.001

*Note*:  ^*∗*^*P*-value derived from one-way analysis of variance (ANOVA). In the retention strength variable, groups with different English letters are statistically different from one another (*P* < 0.05, according to Turkey's post hoc test), so the mean of retention strength in the control group was statistically more than the ceramic and composite groups (*P*=0.006 and *P* < 0.01, respectively), and the mean of retention strength in IPS was statistically more than the ceramic and composite groups (*P*=0.012 and *P* < 0.001, respectively). SD, standard deviation.

## Data Availability

The data used to support the findings of this study are available from the corresponding author upon request.
